# Use of low‐cost three‐dimensional printer to simulate grasping of bronchial foreign body

**DOI:** 10.1002/rcr2.351

**Published:** 2018-07-11

**Authors:** Masayuki Nakayama, Shinichi Yamamoto, Naoki Kaneko, Naoko Mato, Takuji Suzuki, Koichi Hagiwara

**Affiliations:** ^1^ Division of Pulmonary Medicine, Department of Medicine Jichi Medical University Shimotsuke Tochigi Japan; ^2^ Department of General Thoracic Surgery Jichi Medical University Shimotsuke Tochigi Japan; ^3^ Division of Interventional Neuroradiology Ronald Reagan UCLA Medical Center Los Angeles USA

**Keywords:** Bronchial foreign body, three dimensional printer, simulation training, forceps

## Abstract

An 89‐year‐old man was hospitalized with severe pneumonia. Chest computed tomography showed a foreign body in the left main bronchus. We moulded a three‐dimensional (3D) model of the foreign body with a low‐cost 3D printer and found it had the shape of a tooth. We simulated grasping the model with several forceps and succeeded in grasping it with a retrieval net and shark‐tooth forceps. Bronchoscopy was performed after his respiratory condition improved. We found a dental foreign body that had accidentally moved and become stuck in the right upper bronchus. We scraped it out with a retrieval net and grasped the recess site of the foreign body with a shark‐tooth forceps as performed in the simulation. Removal was successful, and the total bronchoscopy time was 9 min. The bronchial foreign body was safely grasped and removed in a short time after simulation with several forceps using a low‐cost 3D printer.

## Introduction

When removing a bronchial foreign body by flexible bronchoscopy, it is important to choose the forceps or device most suitable for the shape and hardness of the foreign body and to grasp it precisely. Removal must be performed in a short time, especially in older patients and those with respiratory failure. However, the identification of the foreign body before bronchoscope insertion is often difficult.

Three‐dimensional (3D) printing is a promising new technology for medical education development and can create sophisticated patient‐specific models from imaging databases. Because their price has recently decreased, 3D printers costing less than USD $3000 have been used in medical simulation training. A cerebrovascular 3D model was created using a low‐cost 3D printer and was used for preoperative simulation 1, and a 3D training model of a central bronchus has also been reported 2.

Here, we report a case involving the simulated grasping and removal of a bronchial foreign body with several forceps using a low‐cost 3D printer.

## Case Report

An 89‐year‐old man with dementia was hospitalized in our department with severe pneumonia. Chest computed tomography (CT) showed left lower lobe consolidation and a small high‐density lesion suspected to be a foreign body in the left main bronchus. We performed bronchoscopy one week after initiation of antibiotic treatment, and his oxygen demand decreased from 7 to 4 L/min.

Before bronchoscopy, we moulded a 3D model of the bronchial foreign body using a low‐cost 3D printer (UP! Plus2; OPT, Tokyo, Japan) with an acrylonitrile butadiene styrene (ABS) resin filament. It took approximately 1 h from uptake of the Digital Imaging and Communications in Medicine chest CT images to the completion of the 3D model. We found that the model had the shape of a molar tooth. Because this 3D printer utilizes a fused filament fabrication (FFF) prototyping system, the surface of the model becomes moderately rough. Therefore, we dipped the model in the ABS solvent eSolve (Kaneko Chemical, Saitama, Japan), a halogen alkylate, to make the surface smoother, resembling a real tooth 3.

**Figure 1:**
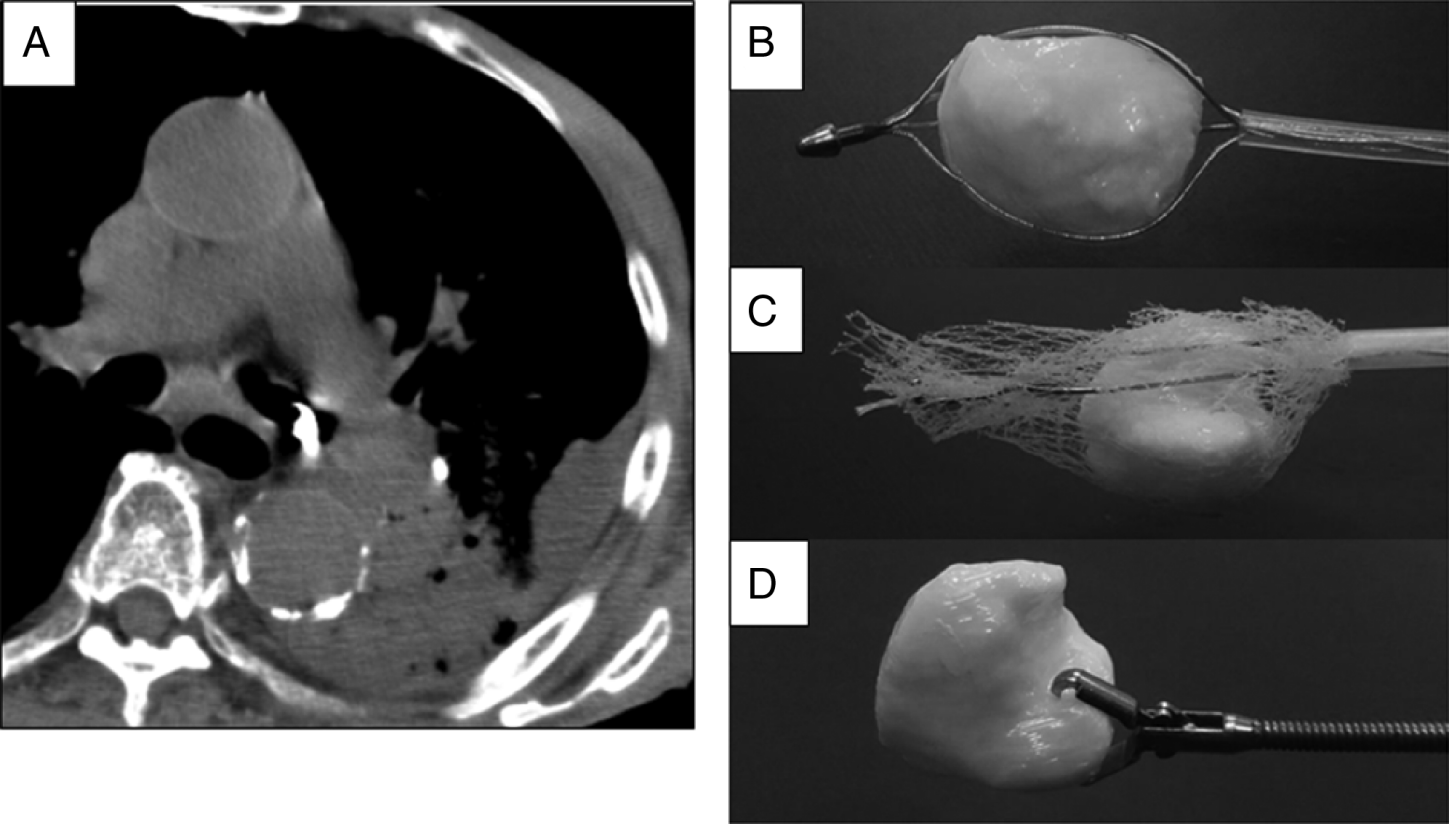
(A) Computed tomography showed a bronchial foreign body in the left main bronchus. We grasped a three‐dimensional model of the foreign body with (B) basket‐type forceps, (C) retrieval net, and (D) shark tooth forceps.

Using this 3D model, we simulated grasping of the foreign body with all available types of grasping forceps. Although we failed to grasp it with several types of biopsy forceps, an alligator forceps, and a five‐pronged grasping forceps, we succeeded using a basket‐type forceps, a retrieval net, and a shark‐tooth forceps, especially when grasping the recess site of the 3D model (Fig. 1).

**Figure 2:**
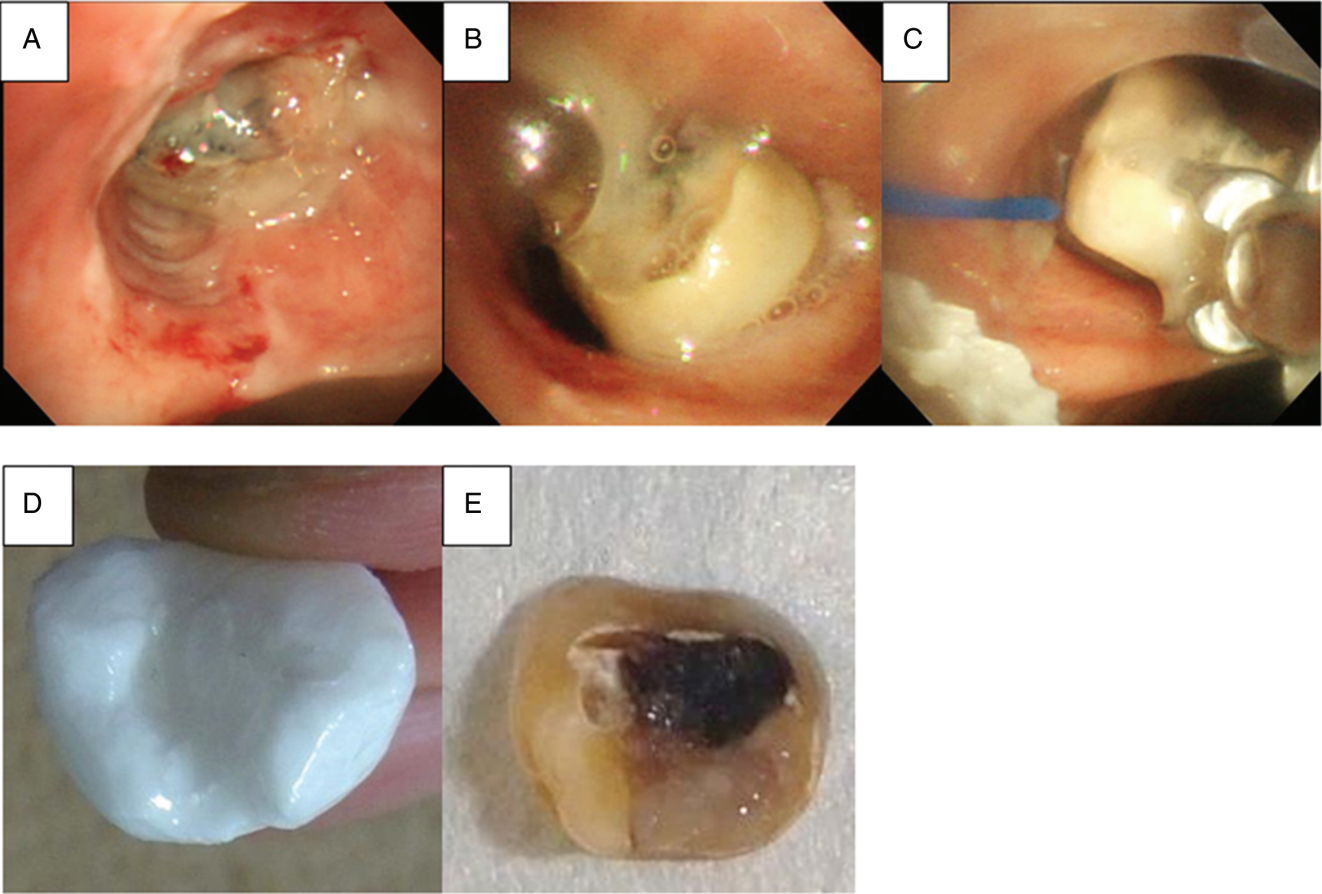
(A) A dental foreign body was stuck in the right upper bronchus. (B) After scraping it out, we recognized the recess site of the foreign body in the truncus intermedius. (C) We grasped and removed it with a shark tooth forceps. (D) A three‐dimensional model was moulded with a low‐cost three‐dimensional printer, and (E) the dental foreign body was removed.

Because space was present around the foreign body in the left main bronchus on chest CT, we initially planned to use a basket‐type forceps or a retrieval net. However, several days after planning, a chest radiograph showed that the foreign body had moved to the right bronchus. When inserting the bronchoscope, a dental foreign body covered with mucinous sputum was stuck in the right upper bronchus. Because the recess site of the foreign body was turned to the peripheral side, we scraped it out to the truncus intermedius using a retrieval net. We could recognize the recess site and thus grasped and removed it with a shark‐tooth forceps as performed in the simulation (Fig. 2). It took just 9 min from bronchoscope insertion to foreign body removal, without adverse events.

## Discussion

The most common bronchial foreign bodies in adults are metallic objects, organic objects, and teeth. Tooth or alligator forceps are usually used to remove flat, thin inorganic, or hard organic foreign bodies. Conversely, a retrieval net or basket‐type forceps is often used to remove soft foreign bodies, especially when space is present around the foreign body 4.

The identification of the foreign body is difficult before bronchoscopy, as is promptly choosing the most suitable forceps and precisely grasping the foreign body during bronchoscopy. We thought that we could quickly remove the foreign body if we could identify it in advance and simulate grasping it with several types of forceps.

We recognized that the 3D model was tooth‐shaped after moulding with the low‐cost 3D printer UP! Plus2 (USD $1300). The moulding time was just 1 h. The FFF system utilized by most low‐cost 3D printers creates stair‐like layers of about 150 μm on the surface of the model. Because the ABS solvent makes the surface of the ABS resin smoother by dipping for only 10 sec, we dipped the 3D model into this solvent.

Based on the patient’s chest CT finding, we initially planned to use a basket‐type forceps. After planning, however, the position and direction of the foreign body changed. Choosing the most effective forceps is sometimes difficult. However, we were able to choose the most appropriate forceps (shark tooth forceps) to grasp the same recess site of the foreign body as performed in the simulation.

The 3D model of the foreign body produced by the low‐cost 3D printer in this study allowed us to understand the actual shape of the foreign body, test all available forceps and devices, determine which forceps we could and could not use, and determine the optimal site for grasping the foreign body. This simulation enabled safe and quick foreign body removal in a high‐risk patient.

In conclusion, we safely removed a foreign body in a short time through the simulation of grasping it with several types of forceps using a low‐cost 3D printer.

### Disclosure Statement

Appropriate written informed consent was obtained for publication of this case report and accompanying images.
